# Effect of 60 kHz and 150 kHz Femtosecond Lasers on Corneal Stromal Bed Surfaces: A Comparative Study

**DOI:** 10.1155/2013/971451

**Published:** 2013-08-29

**Authors:** Cristina Monterosso, Alessandro Galan, Elisabetta Böhm, Alfonso Zampini, Mohit Parekh, Luigi Caretti

**Affiliations:** ^1^Department of Ophthalmology, Dell'Angelo Hospital, Mestre, 30174 Venice, Italy; ^2^Department of Ophthalmology, S. Antonio Hospital, S. Paolo Centre, Padua, 35127 Venice, Italy; ^3^The Veneto Eye Bank Foundation, Via Paccagnella 11, Mestre, 30174 Venice, Italy

## Abstract

*Purpose*. To compare the effect of 60 kHz and 150 kHz femtosecond (FS) laser on the corneal stromal bed surfaces (SBS). *Methods*. Sixteen human donor corneal tissues unsuitable for transplantation were used. Anterior and posterior lamella was obtained using 60 kHz and 150 kHz FS laser. A standard depth of 400 **μ**m was set for anterior lamellar keratoplasty (ALK) and endothelial lamellar keratoplasty (ELK). The quality and smoothness of the SBS post-FS laser dissection were graded for statistics. *Results*. No intraoperative complications were found. The side cuts were straight, and the SBS appeared smoother in cuts obtained using 150 kHz. The average values of the SBS quality of the anterior lamellar cut were found to be 2.25 (±0.28) for 60 kHz and 3.125 (±0.25) for 150 kHz (*P* = 0.0039). Whereas, 2 (±0.4) for 60 kHz and 2.75 (±0.28) for 150 kHz (*P* = 0.0273) was the quality observed in endothelial cuts. No significant difference was found between anterior and posterior cuts performed using the same FS laser (60 kHz or 150 kHz) (*P* > 0.05). *Conclusions*. The 60 kHz and 150 kHz FS lasers are equally effective in performing lamellar dissection for ALK and ELK. 150 kHz FS laser allows a tighter spot and layer separation which creates a slightly smoother SBS.

## 1. Introduction

Corneal lamellar keratoplasty (LK) is a surgical technique that allows preserving healthy portions of the cornea while selectively replacing the dysfunctional layers. The outcome of LK has also shown improvements of the procedure by decreasing surgical risk, enhancing healing process and quick rehabilitation as compared to penetrating keratoplasty (PK) [1]. Since the last decade, the femtosecond (FS) laser technology has been developed to perform laser assisted in situ keratomileusis (LASIK) refractive surgery. It reduced the complication rate due to LASIK flap creation, improved the predictability of flap dimensions, and improved the quality of the optical surface, compared to microkeratome surgery [2–8]. The reliability and safety have been evaluated using IntraLase FS (iFS, Abbott, Irvine, CA, USA) laser which have already been verified extensively for LASIK and more recently for endothelial lamellar keratoplasty (ELK) [9, 10]. The FS uses pulses to create corneal resection. The quality of the surfaces obtained is determined by programmable parameters like the laser spot and layer separation and the energy delivered per pulse. It was therefore evaluated that, with closer spots, the energy required for the cuts is less and with less energy surface gets smoother [2]. 

The early stage of this technology allowed the firing rate of 2 kHz, spot and layer separation of 14/14, and the energy per spot of 6 *μ*J. The 60 kHz iFS was introduced in 2006; the minimum spot and layer separation that can be used with this laser for a lamellar cut is 4/4, and the energy can be set from 0.9 to 1.1 *μ*J. In 2009 the 150 kHz iFS technology was introduced. The speed of laser spot delivering further increased which allows a spot and layer separation of 6/6 and an energy level setting between 0.6 and 0.8 *μ*J for LASIK surgery, a spot and layer separation of 2/2 and an energy level setting between 0.75 and 1 *μ*J for deep lamellar surgery. Thus, with time, the quality of the cuts gradually increased with higher engine speed creating a tighter spot and line separation with lower energy per spot giving rise to smooth and efficient cuts [6, 7]. In the recent years, the FS laser has been used to perform lamellar corneal surgery. Some studies reported the use of the FS laser to create donor tissue for ELK [9–14]. Others showed good results with anterior lamellar keratoplasty (ALK) [15, 16]. The purpose of this study therefore was to compare the quality of the stromal corneal surface obtained with a 60 kHz and a 150 kHz iFS laser, cut at the same depth to perform both ALK and ELK.

## 2. Materials and Methods

### 2.1. Cut Creation

**Figure 1:**
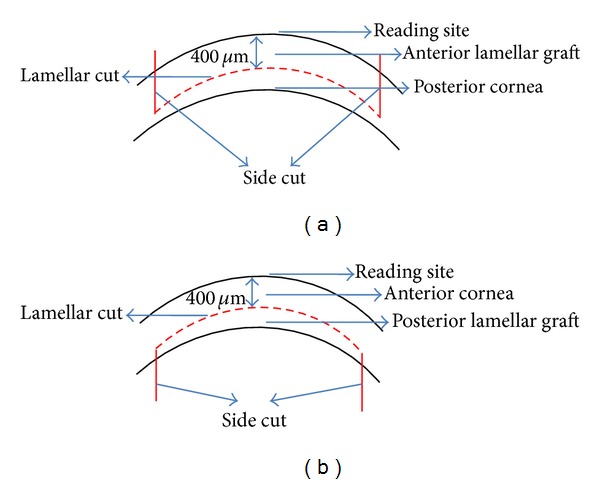
IntraLase FS laser reading site and cut site. The IntraLase FS laser measures the cut site from epithelium. Thus a standard depth of 400 *μ*m was maintained both for anterior and posterior cuts. (a) Since the FS laser begins the cut from the endothelium, anterior lamellar graft was prepared by applying lamellar cut first followed by the vertical or side cut. The thickness was maintained at 400 *μ*m from the anterior reading site. (b) In posterior lamellar graft the side cut is performed first followed by the lamellar cut. The thickness was maintained at 400 *μ*m from the anterior reading site. The posterior lenticule retains its thickness in the range of 150 ± 40 *μ*m depending on initial corneal thickness.

Sixteen organ cultured donor corneas (unsuitable for transplantation) with complete intact epithelial layer, without any stromal opacities and approximately 2 mm scleral rim, were obtained from the Veneto Eye Bank Foundation. Corneas were preserved at 31°C in organ culture followed by 24 hours of storage in deturgescence medium containing 6% dextran T500 (deswelling agent). Eight donor corneas were cut using 60 kHz iFS and the other eight using 150 kHz iFS. Four anterior and four posterior lamellar and side cuts were performed using both iFS by two surgeons (C. M., L. C.). The donor corneas were mounted on an artificial anterior chamber (Moria, Anthony, France) and were dissected with the FS laser. iFS parameter settings included lamellar depth of 400 *μ*m, side cut angle 90°, trephination diameter 8.5 mm, energy for the lamellar cut 1 *μ*J with a 2/2 spot and layer separation for the 150 kHz FS laser, and 0.9 *μ*J with a 4/4 spot and layer separation for the 60 kHz FS laser. The optimum energy level settings were obtained performing preliminary cuts in order to reach the best surface outcome for each laser with the lowest energy delivery (performed on separate corneas). In all cases the stromal interface was evaluated on the residual stromal bed attached to the sclera. The laser performs a posterior side cut first and then lamellar for the endothelial disc and lamellar first followed by anterior vertical cut for the anterior disc, since the laser begins the cut from the endothelium and comes up towards the epithelium (Figure 1). The tissues were separated by the surgeons lifting the lamella with a corneal forceps with the help of a blunt spatula used to counter without sweeping across the interface.

### 2.2. Scanning Electron Microscopy

Corneoscleral bed and lenticules were immersed in 2.5% glutaraldehyde and sent to the laboratory for Scanning Electron Microscopy (SEM) immediately. Samples were rinsed in phosphate-buffered solution and dehydrated in ascending concentrations of ethanol. These samples were further treated with liquid CO_2_ at 0°C to 5°C and then at 32°C in a critical point drier (CPD030 BAL-TEC AG, Balzers, Liechtenstein) which was later attached to aluminum SEM stubs with graphite adhesive tape and sputter coated with gold. The specimens were viewed on a JSM 6490 SEM (Jeol Ltd., Tokyo, Japan) to observe the side cut and the appearance of the dissection on the corneal stromal bed at different magnifications.

### 2.3. Grading

The images obtained by SEM were randomly numbered and assessed by two blinded observers (A. Z., E. B.), not involved in laser procedures. The samples were presented first to compare the anterior lamellar cuts followed by posterior lamellar cuts and finally to compare anterior and posterior lamellar cuts obtained with the same repetition rate (iFS 60 and 150 kHz) in order to appreciate if the timing of the laser side cut versus lamellar cut could influence the quality of the stromal bed. The grading was carried out at about 30x and 250x magnification. The observers graded the stromal bed quality on a scale of 1–4 (4 corresponds to smooth, 3.5 to mild rough to smooth, 3 to mild rough, 2.5 to moderate rough to mild rough, 2 to moderate rough, 1.5 to rough to moderate rough, and 1 to rough). The grading was carried out at the lowest magnification in order to allow the observer to view the whole cut. The observers graded the stromal bed quality considering that the smoothest SBS was the closer to the near uncut epithelium (for ALK) or Descemet (for ELK).

### 2.4. Statistical Analysis

Student's *t* test was used to determine the statistical significance of mean differences of the grading results between 60 kHz and 150 kHz cuts and between cuts performed by the same repetition rate. Analyses were conducted with SAS version 9.2 statistical software (SAS Institute Inc., Cary, NC, USA). The subjective analysis converted to objective values was compared for each group of the corneas, that is, anterior SBS analysis cut using iFS 60 kHz versus iFS 150 kHz, posterior SBS analysis cut using iFS 60 kHz versus iFS 150 kHz, and finally anterior versus posterior cuts with the same laser. 

## 3. Results

**Figure 2:**
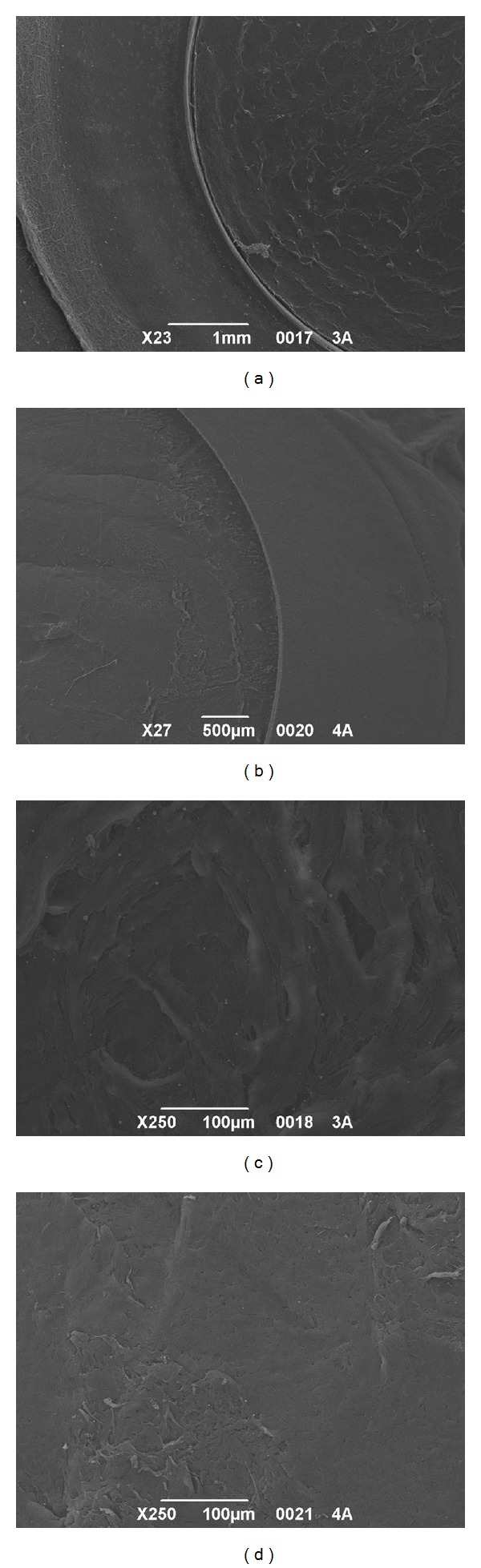
SEM comparison of corneal stromal bed after iFS 60 kHz and 150 kHz delivery to perform anterior cuts. (a) Exposed stromal bed after the 60 kHz laser delivery at 23x magnification. (b) Exposed stromal bed after the 150 kHz laser delivery at 27x magnification. (c) Central stromal bed at 250x magnification after the 60 kHz laser delivery. (d) Central stromal bed at 250x magnification after the 150 kHz laser delivery.

**Figure 3:**
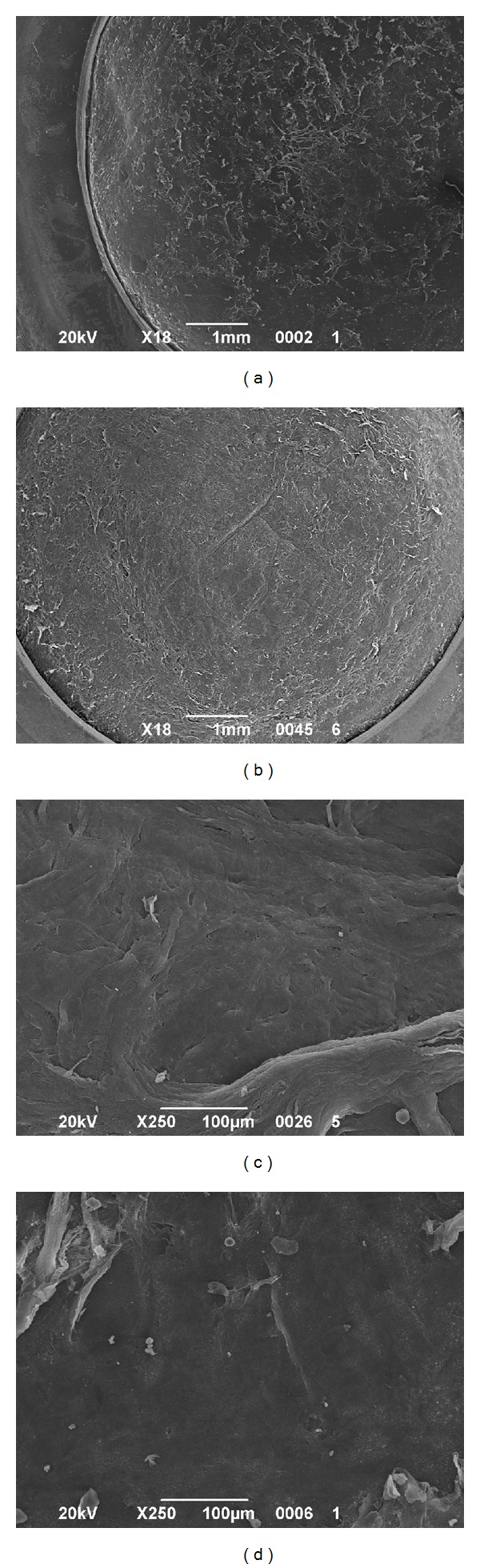
SEM comparison of corneal stroma after the 60 kHz and 150 kHz iFS delivery to perform posterior cuts. (a) Scleral rim and exposed stromal bed after iFS 60 kHz laser delivery at 18x magnification. (b) Scleral rim and exposed stromal bed after the 150 kHz laser delivery at 18x magnification. (c) Central stromal bed at 250x magnification after the 60 khz laser delivery. (d) Central stromal bed at 250x magnification after the 150 khz laser delivery.

**Figure 4:**
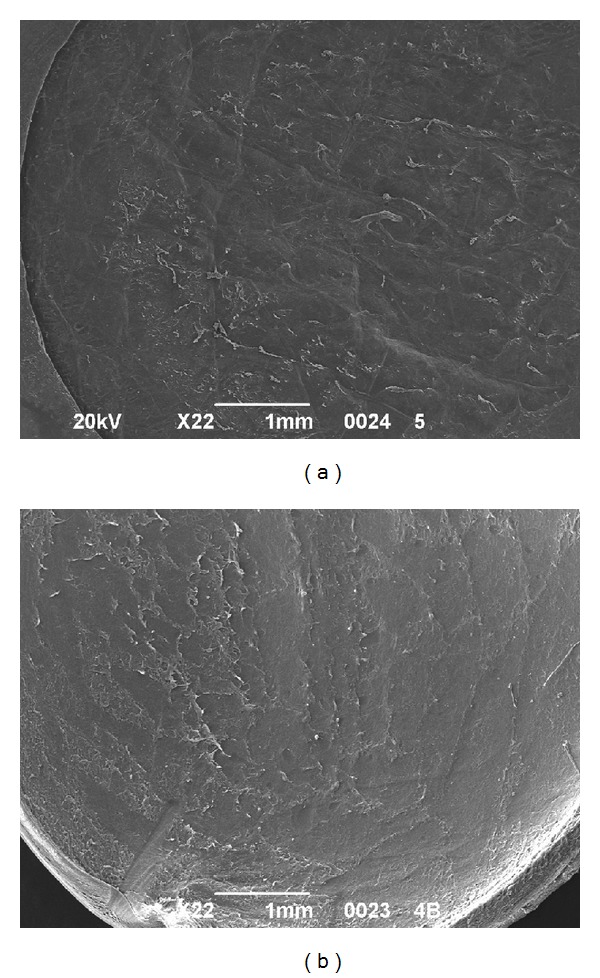
SEM analysis of corneal SBS after cutting with 60 kHz iFS. (a) Anterior cut at 22x magnification. (b) Endothelial cut at 22x magnification.

**Figure 5:**
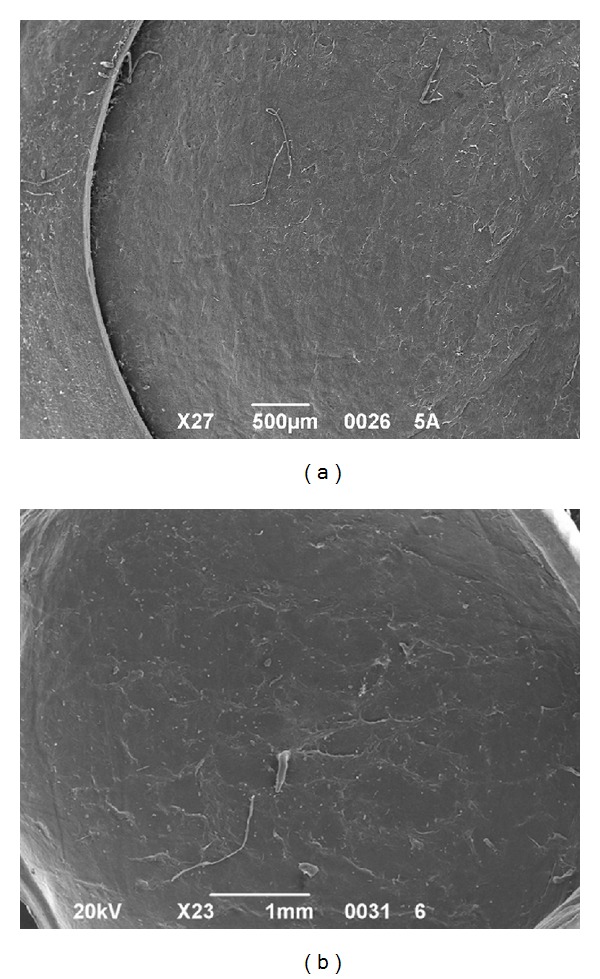
SEM analysis of corneal SBS after cutting with 150 kHz iFS. (a) Anterior cut at 27x magnification. (b) Endothelial cut at 23x magnification.

All the samples were successfully cut using iFS 60 and 150 kHz lasers without any intraoperative complications. All the side cuts were straight with no visible damage. The SBS appeared smooth both in 60 kHz and 150 kHz cuts. It was observed that the SBS obtained with iFS 150 kHz was slightly smoother than those obtained with iFS 60 kHz (Figures 2 and 3). The stromal disruptions looked widely spread throughout the cornea with 60 kHz iFS, whereas less disruptions were observed using 150 kHz iFS. A mild stucco-like texture was observed in a few posterior samples both in the 60 kHz and 150 kHz iFS groups (Figures 2 and 3). With both repetition rates, a smoother cut surface was observed on the anterior specimens than the posterior (Figures 4 and 5).

**Figure 6:**
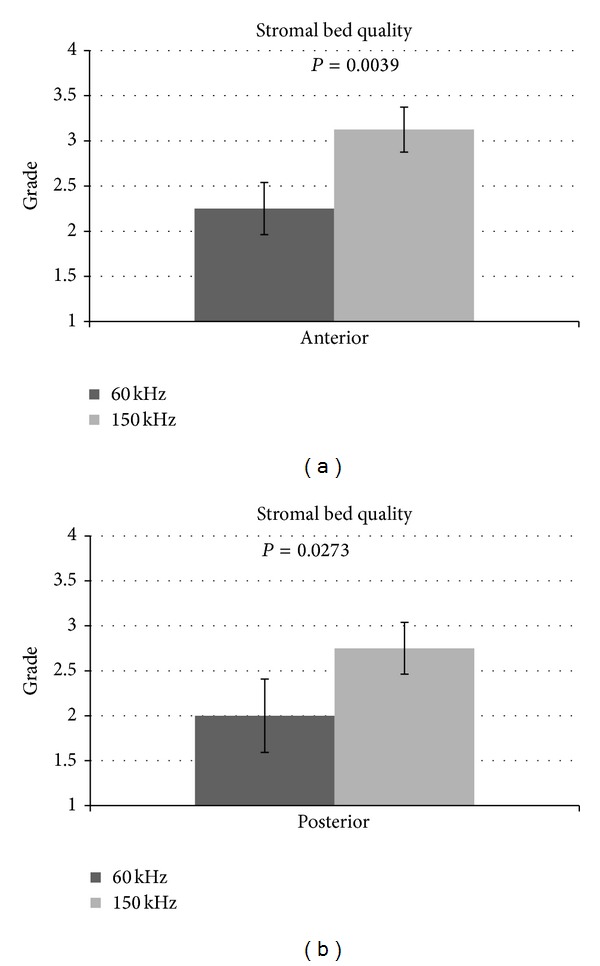
(a) Comparison of anterior stromal bed quality using both iFS 60 kHz and 150 kHz. (b) Comparison of posterior stromal bed quality using both iFS 60 kHz and 150 kHz.

**Figure 7:**
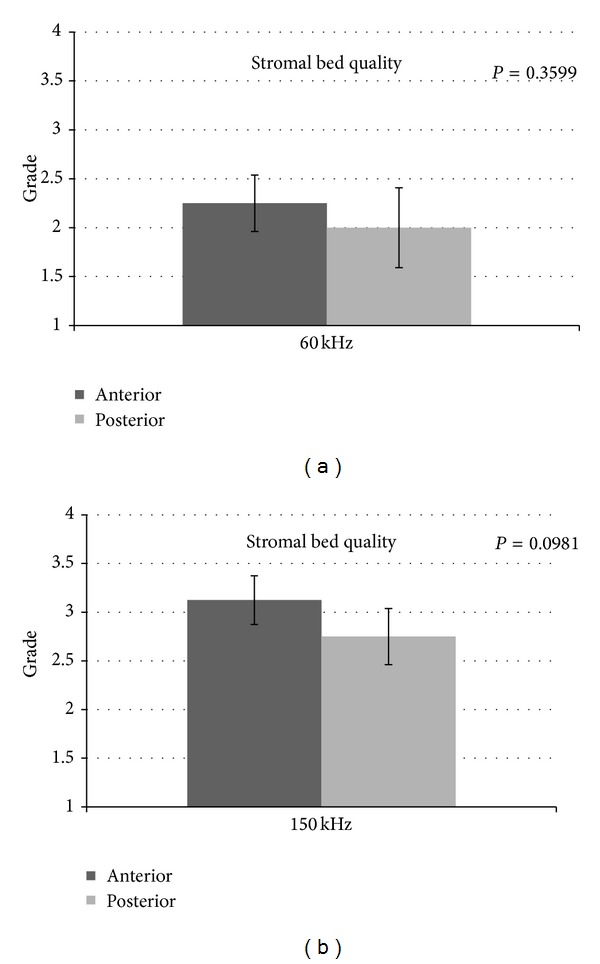
(a) Comparison of anterior and posterior stromal bed quality using iFS 60 kHz. (b) Comparison of anterior and posterior stromal bed quality using iFS 150 kHz.

As iFS reads the thickness from the epithelial side, the anterior lamellar graft was always 400 *μ*m thick, whereas the posterior lamellar graft was found normally in the range of 150 ± 40 *μ*m thick depending on the overall thickness of the cornea. The time to create the lamellar dissections with iFS 150 kHz in comparison to iFS 60 kHz was almost half. The mean values of the anterior stromal bed quality was 2.25 (±0.28) for 60 kHz and 3.13 (±0.25) for 150 kHz (*P* = 0.0039) (Figure 6(a)). Posterior stromal bed quality was 2.00 (±0.41) for 60 kHz and 2.75 (±0.28) for 150 kHz (*P* = 0.0273), as shown in Figure 6(b). The results indicated that the anterior stromal surface cut using iFS 150 kHz was the smoothest amongst all the cuts performed, and the posterior stromal bed cut using 60 kHz iFS was the roughest amongst all the cuts performed. Also, the anterior and posterior SBS were smoother when the corneal tissue was cut using 150 kHz iFS as compared to 60 kHz iFS. There was no statistically significant difference observed with anterior or posterior cuts using the same iFS laser (*P* > 0.05) as shown in Figures 7(a) and 7(b).

## 4. Discussion

LK is a valid alternative to perforating keratoplasty because it allows replacing the damaged portion of the cornea. Deep anterior lamellar keratoplasty (DALK) is the first choice of surgery for anterior corneal stromal pathologies [15]. ALK is a tough alternative in selected patients with stromal opacities limited to the anterior stroma and is more predictable than anterior descemetic keratoplasty. In the last years, ELK evidenced its advantages over traditional keratoplasty offering an overall prevention to high irregular astigmatism.

The use of the FS laser to perform LK was evaluated in several in vitro and animal studies [17, 18]. In 2007 Cheng et al. first reported an FS laser-assisted ELK, preparing the donor cornea with the FS laser [19]. In a recent study using SEM, the residual donor stroma of an endothelial disc prepared for ELK after a 60 kHz FS laser resulted in a smooth surface with a precise side cut [11]. It has been shown that stromal bed quality can be improved by using lower pulse energy and spot separation settings [2]. Soong et al. [20] found that a 15 kHz FS laser produced posterior lamellar cuts with a mild stucco-like texture of the interface on SEM. They postulated that it might be caused due to increased scatter and attenuation of laser energy at the deeper cut settings and by the looser lamellar fibrillar configuration of deep stroma. They also observed that the posterior stroma may be susceptible to circular wrinkling induced by the corneal applanation that reflects the circular ridges seen on the stromal bed after the cut. In our samples we noticed these circular wrinkling only in the 60 kHz group.

Sarayba et al. [2] demonstrated that a 30 kHz FS laser creates a smoother LASIK surface than a 15 kHz FS laser. The 60 kHz FS laser allows closer spot separation with lower energy levels and results in smooth interface also in deeper cuts. The closer the spots are, the easier the lift of the lamella will be, because the tissue bridges are reduced. 

The 150 kHz FS technology further reduced the gap between the spots, creating in our series a slightly smoother stromal surface. In our series we observed a mild stucco-like texture of the stromal surface only in some samples at high magnification, and this relates to the low rate of tissue bridges we found with both lasers. This is confirmed by the ease of lifting the amputated lenticule, without sweeping a spatula across the interface but just using it to push down the stromal bed. With the 150 kHz repetition rate, the laser delivery time significantly increased; this allows shorter suction application, decreasing the time of high intraocular pressure and decreasing the risk of suction breaks that mostly tends to occur after 30 seconds of suction application [3].

In our study we also compared anterior with posterior lamellar and side cuts performed with the same repetition rate to verify whether the sequence of creation of the laser cut could highlight some difference in smoothness. The laser performs posterior vertical cut first and then lamellar for the ELK and lamellar first and then anterior vertical cut for the ALK. We noticed for both iFS lasers that the interface was better in anterior samples (Figures 3 and 4). Our experience showed that this could be a possibility that is related to the bubble gas escape through posterior vertical cuts during the lamellar cut when the endothelial disc is prepared, making the photo-disruption process of the FS laser less effective.

In our opinion, the donor corneas for endothelial surgery can be prepared creating only the horizontal cut at the programmed depth to obtain a smoother interface. Then the cornea can be punched in again with the endothelial side up to prepare an endothelial lenticule of the desired diameter. In conclusion, we documented that both 60 kHz and 150 kHz FS lasers are effective in creating lamellar dissection for lamellar keratoplasty. The smoothness of the interface has been documented by the SEM analysis. The new 150 kHz femtosecond laser permits a closer spot and line separation showing a slightly smoother corneal stromal bed.
